# Effect of Personalized Incentives on Dietary Quality of Groceries Purchased: A Randomized Crossover Trial

**DOI:** 10.1001/jamanetworkopen.2020.30921

**Published:** 2021-02-10

**Authors:** Maya Vadiveloo, Xintong Guan, Haley W. Parker, Elie Perraud, Ashley Buchanan, Stephen Atlas, Anne N. Thorndike

**Affiliations:** 1 Department of Nutrition and Food Sciences, College of Health Sciences, University of Rhode Island, Kingston; 2 Marketing Area, College of Business Administration, University of Rhode Island, Kingston; 3 AgroParis Tech, Paris, France; 4 Department of Pharmacy Practice, College of Pharmacy, University of Rhode Island, Kingston; 5 Division of General Internal Medicine, Department of Medicine, Massachusetts General Hospital, Boston; 6 Harvard Medical School, Boston, Massachusetts

## Abstract

**Importance:**

Many factors are associated with food choice. Personalized interventions could help improve dietary intake by using individual purchasing preferences to promote healthier grocery purchases.

**Objective:**

To test whether a healthy food incentive intervention using an algorithm incorporating customer preferences, purchase history, and baseline diet quality improves grocery purchase dietary quality and spending on healthy foods.

**Design, Setting, and Participants:**

This was a 9-month randomized clinical crossover trial (AB–BA) with a 2- to 4-week washout period between 3-month intervention periods. Participants included 224 loyalty program members at an independent Rhode Island supermarket who completed baseline questionnaires and were randomized from July to September 2018 to group 1 (AB) or group 2 (BA). Data analysis was performed from September 2019 to May 2020.

**Intervention:**

Participants received personalized weekly coupons with nutrition education during the intervention period (A) and occasional generic coupons with nutrition education during the control period (B). An automated study algorithm used customer data to allocate personalized healthy food incentives to participant loyalty cards. All participants received a 5% grocery discount.

**Main Outcomes and Measures:**

Grocery Purchase Quality Index–2016 (GPQI-16) scores (range, 0-75, with higher scores denoting healthier purchases) and percentage spending on targeted foods were calculated from cumulative purchasing data. Participants in the top and bottom 1% of spending were excluded. Paired *t* tests examined between-group differences.

**Results:**

The analytical sample included 209 participants (104 in group 1 and 105 in group 2), with a mean (SD) age of 55.4 (14.0) years. They were predominantly non-Hispanic White (193 of 206 participants [94.1%]) and female (187 of 207 participants [90.3%]). Of 161 participants with income data, 81 (50.3%) had annual household incomes greater than or equal to $100 000. Paired *t* tests showed that the intervention increased GPQI-16 scores (between-group difference, 1.06; 95% CI, 0.27-1.86; *P* = .01) and percentage spending on targeted foods (between-group difference, 1.38%; 95% CI, 0.08%-2.69%; *P* = .04). During the initial intervention period, group 1 (AB) and group 2 (BA) had similar mean (SD) GPQI-16 scores (41.2 [6.6] vs 41.0 [7.5]) and mean (SD) percentage spending on targeted healthy foods (32.0% [10.8%] vs 31.0% [10.5%]). During the crossover intervention period, group 2 had a higher mean (SD) GPQI-16 score than group 1 (42.9 [7.7] vs 41.0 [6.8]) and mean (SD) percentage spending on targeted foods (34.0% [12.1%] vs 32.0% [13.1%]).

**Conclusions and Relevance:**

This pilot trial demonstrated preliminary evidence for the effectiveness of a novel personalized healthy food incentive algorithm to improve grocery purchase dietary quality.

**Trial Registration:**

ClinicalTrials.gov Identifier: NCT03748056

## Key Points

**Question:**

Does a semiautomated, personalized, healthy food incentive intervention improve grocery purchase dietary quality and percentage spending in targeted food groups?

**Findings:**

In this randomized clinical crossover trial of 209 adult participants, healthy food incentives personalized according to customer data were associated with a small but significant improvement in grocery purchase quality and percentage spending on targeted food groups.

**Meaning:**

A personalized healthy food incentive intervention increased grocery purchase quality and may be a promising population-based strategy for improving dietary intake.

## Teaser

This randomized, clinical crossover trial examines whether a healthy food incentive intervention using an algorithm incorporating customer preferences, purchase history, and baseline diet quality improves grocery purchase dietary quality and spending on healthful foods.

## Introduction

Improving population-level dietary quality is essential to reduce the burden of diet-related chronic diseases.^1^ Most US adults’ diets achieve 60% of Dietary Guidelines designed to promote health^2,3^ partly because food choice is associated with numerous factors, including taste, availability, food marketing, and nutrition knowledge.^4^ Moreover, system-level barriers, including nutrition misinformation, choice overload, and cost, impede healthy eating patterns.^5^ Multicomponent interventions to improve food choice extending beyond nutrition education^6^ have become more common with greater recognition of the complexity of food decisions.^7,8,9,10^ Insights from behavioral economics, including choice architecture, taxation, and subsidies, have enhanced behavioral interventions and demonstrated short-term success toward improving health behaviors.^11^ Collectively, this paradigm shift accentuates the importance of developing novel interventions that address individual- and system-level barriers to enhance dietary decisions.

Results from several grocery store–based trials^12,13,14^ bolster an optimistic view about the promise of interventions targeting healthier purchasing behavior. Most studies have focused on increasing fruit and vegetable intake^15,16,17,18,19^ and support using coupons or incentives to improve diet quality.^20^ Microsimulation studies^21^ estimate that improving fruit and vegetable intake among participants receiving nutrition assistance could save $1.2 billion in health care costs. Limited research has found sustained changes to purchasing behaviors after incentives are discontinued,^22^ suggesting that incentives may encourage new dietary habits. Nevertheless, research targeting a wider variety of healthy foods has been more variable.^12,13,14^

Personalization, compared with one-size-fits-all approaches,^23,24^ may enhance the success of dietary interventions. A 6-month trial in New Zealand^12^ found that a discount on healthier foods (>1000 products) based on participants’ usual purchasing habits and brand preferences modestly increased healthy food purchases. Conversely, a South African trial^25^ found no effect of personalized discounts on increasing healthy food purchases. Companies like Amazon.com, Inc apply data analytics to personalize recommendations,^26^ and food marketers send personalized coupons to increase customers’ food purchases.^27^However, machine-learning algorithms have not been explored as a scalable strategy to personalize healthy dietary recommendations to improve dietary quality. The objective of the Smart Cart randomized clinical crossover trial was to examine the preliminary effectiveness of using grocery purchase data and other individual-level diet-related metrics to provide semiautomated personalized incentives to increase overall grocery purchase quality and percentage spent on targeted foods.

## Methods

### Study Design

The Smart Cart Study was a 9-month, randomized, clinical crossover trial (AB–BA design) testing the effect of personalized weekly incentives on grocery purchase quality and percentage spending on incentivized healthy food groups. The crossover design was selected to enhance our ability to recruit community-based participants and increase statistical power. Institutional review board approval was obtained at the University of Rhode Island on May 30, 2018; the trial protocol is included in Supplement 1. A complete description of the study design and theoretical underpinnings for the Smart Cart Study has been previously described.^28^ This study followed the Consolidated Standards of Reporting Trials (CONSORT) reporting guidelines for a crossover trial.^29^

### Participants and Setting

The Smart Cart Study^28^ was conducted with 1 independent grocery retailer in Rhode Island. Participant recruitment occurred between July and September 2018. Eligible participants were aged 18 years or older, English-speaking, primary household shoppers, not pregnant, purchased 50% or more of their groceries with the supermarket, and willing to use the store’s loyalty card and receive weekly emails. Participants completed a written informed consent form and 2 baseline questionnaires and were subsequently randomized to 1 of 2 groups: group 1 (AB) underwent a 13-week intervention and a 2- to 4-week washout period, followed by a 12-week control period, and group 2 (BA) underwent a 13-week control period and a 2- to 4-week washout period, followed by a 12-week intervention. The study statistician (A.B.) independently randomized participants using blocked randomization (28 blocks, size 8, for a total of 224 participants).

### Intervention Design

Development of the study intervention has been described elsewhere.^28^ Briefly, before participant recruitment, the study team analyzed 1 year of store-level purchasing data (January 2017 to January 2018) to develop the healthy coupon algorithm. Commonly purchased foods were identified from their Universal Product Code (UPC) and description and categorized into food groups, and the publicly available Guiding Stars search tool was used to evaluate the healthfulness of individual foods and identify possible within-food group healthier substitutes. Foods with less healthy Guiding Stars ratings (ie, 0 or 1 star) were categorized as trigger foods if a healthier alternative (ie, 2 or 3 stars) was available within the same food group; coupons were developed for these healthier alternatives across target food groups. Brief nutrition education messages related to identified healthier alternatives were also developed to link with those coupons. This process allowed for detection of trigger foods from study participants’ purchasing data during the trial. Coupons (141 in total) were developed within low-fat dairy and dairy alternatives, whole grains, nuts, soy or plant-based proteins, lean meat, poultry, fish and shellfish, unsweetened beverages, and produce. Each coupon could be applied to approximately 5 products within the food group, covering approximately 1342 products, 65% of which were produce.

The study team subsequently developed an adaptive relational database to organize this information so that individual-level daily purchase data could be categorized and trigger foods could be identified.^28^ This information was also filtered through individual dietary preferences, food restrictions, and areas for diet quality improvement before personalized coupons aligned with those parameters were selected.

Participants were recruited after the relational database development (July to September 2018), and the 2 baseline questionnaires were used as input into the relational database. The first questionnaire was an online, validated^30^ food frequency questionnaire (FFQ) developed by VioCare^31^ to assess diet quality on the basis of the previous 3 months of self-reported intake, and the second was a sociodemographic, food, and health behavior questionnaire administered online using RedCap. The FFQ generated a validated Healthy Eating Index (HEI)–2010^32^ overall diet quality score at baseline to identify areas for dietary improvement for each participant. The FFQ was readministered during the washout period, and both the FFQ and RedCap questionnaire were administered at study completion.

After randomization, participant-linked purchasing data were automatically collected through loyalty cards. Daily sales data were sent to the study team and included the loyalty card identification number, UPC, and text descriptions of each food, the unit price, number of units purchased, date of the transaction, coupon usage, and total spent. The study team categorized monthly sales data into food groups used to calculate grocery purchase quality and to examine spending in targeted food groups.

### Intervention and Control Groups

All participants received a 5% discount for using their loyalty cards (maximum of $25 per month) as a general incentive to ensure tracking of grocery purchases and intervention delivery. During the intervention period for each group, participants received weekly emails that provided brief nutrition education and 2 personalized coupons ($10 value) for foods that would improve their diet quality and reflected their dietary preferences. Coupon format varied and included percentage discounts, buy-one-get-one free, and dollars off a purchase with 2- to 4-week expiration dates. Personalized weekly emails might contain a coupon for plain yogurt, brief information about the benefits of calcium, and a yogurt parfait recipe. Coupons were selected on the basis of the adaptive relational database, which was updated weekly with participant’s weekly purchases and monthly Grocery Purchase Quality Index–2016 (GPQI-16) score. Coupons were linked to participants’ loyalty cards automatically and were redeemed when the card was scanned at the beginning of the transaction; therefore, participants did not need to display coupons to the cashier. During the control period for each group, participants received weekly emails with generic, brief nutrition education and a coupon for $2 off their purchase monthly.

### Outcomes and Data Collection

#### GPQI-16 Score and Percentage Spending on Targeted Foods

The GPQI-16 score (range, 0-75 points, with higher scores denoting healthier purchases) was used to measure monthly and period-level grocery purchase quality.^33^ The GPQI-16 compares actual vs recommended spending across food categories and strongly correlates with the validated HEI-2015.^34^ Participants’ daily grocery purchasing records were concatenated, and UPCs were coded into 1 of 13 categories, 11 of which represented GPQI-16 components and 2 which indicated either nonfood items or food items not categorized in the GPQI-16 (eg, oils, condiments, coffee, soups, and mixed dishes). Ratios of component-specific percentages of total spending to recommended spending^35^ were calculated and then multiplied by the maximum component score value (5 or 10 points) and summed to examine overall and component GPQI-16 scores. Food groups where fewer purchases are desirable (ie, refined grains, sodas and sweets, and processed meats) were scored inversely so higher scores reflected a healthier diet. The percentage of spending on targeted foods was computed by dividing spending on couponed UPCs sent to each household by total food spending and household size during each period.

#### Healthy Eating Index–2010

The FFQ^31^ generated HEI-2010 total scores for primary respondents at baseline, washout, and study completion; HEI-2015 scores were not yet available. Change in HEI-2010 scores were evaluated as a secondary outcome and calculated as midpoint HEI-2010 minus baseline HEI-2010 for the initial intervention period and as end-point HEI-2010 minus midpoint HEI-2010 for the crossover intervention period.

### Statistical Analysis

Descriptive statistics for the overall sample and between groups were calculated. An AB–BA crossover analysis was conducted using *t* tests to determine whether GPQI-16 scores and percentage spending on targeted foods differed across study periods and between study groups. Monthly variation in GPQI-16 total and component scores and percentage spending on targeted foods during each period was examined descriptively, without hypothesis testing, to view seasonal trends and food groups responsive to change.

Using a 2-sided *t* test with a 5% type I error rate, assuming the mean (SD) GPQI-16 was similar to 30.26 (6.5), a correlation of 0.70 within participants, and 10% loss to follow-up, the study was powered at 80% to detect a 3% or greater difference in diet quality scores between study groups among the 224 participants. To evaluate changes in the secondary outcome of HEI-2010 scores, participants who reported eating less than 1000 kcal or more than 4500 kcal daily were deemed as having implausible data^30^ and were removed (54 participants with implausible data at any time point). Among the remaining 170 participants, 31 had missing scores either at midpoint or at study completion, leaving 139 unique participants in the complete case analysis. Missing data patterns were evaluated; data were not missing at random and multiple imputation was conducted on baseline, midpoint, and end-point data to generate 20 complete data sets, assuming a multivariate normal distribution.^36,37^ The *t* tests were performed on each imputed data set, and variance was combined using the Rubin estimator before analysis.^38^ Statistical analyses were completed in SAS statistical software version 9.4 (SAS Institute) using 2-tailed tests and an α threshold of *P* < .05. Data analysis was performed from September 2019 to May 2020.

## Results

**Figure 1.**
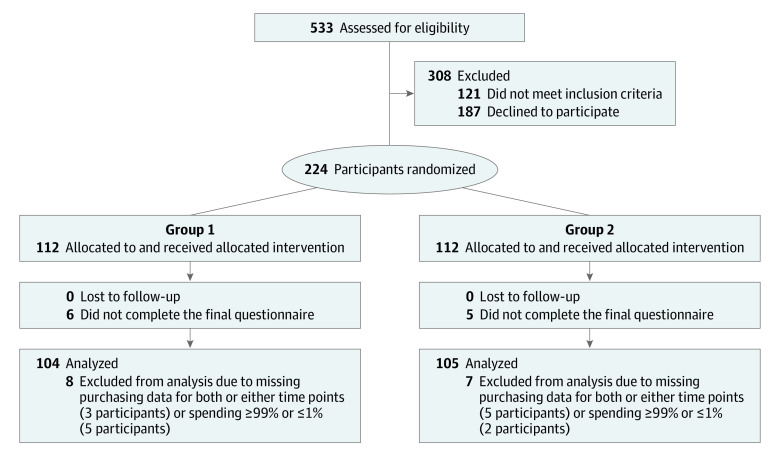
Participant Enrollment Flowchart for the Randomized Clinical Crossover Smart Cart Study Group 1 received the treatment in period 1 followed by an active control in period 2 (AB design). Group 2 received the active control in period 1 followed by the intervention in period 2 (BA design).

From 224 enrolled participants, 209 (93%) participants were included in the analytical sample (104 in group 1 and 105 in group 2). Participants were excluded if they did not make any purchases with the store during 1 or more of the study periods (8 participants). On the basis of the sample distribution, participants whose total spending was outside typical spending during the study period (<1% [$21.67-$37.85], 4 participants, or >99% [$4633-$4832], 3 participants) were removed (Figure 1).

The 209 study participants had a mean (SD) age of 55.4 (14.0) years. The numbers of participants with complete demographic information ranged from 161 to 209. Most participants were women (187 of 207 participants [90.3%]) and non-Hispanic White (193 of 206 participants [94.1%]) (Table 1). One hundred two of 208 participants (49.0%) had a bachelor’s degree or higher, 81 of 161 participants (50.3%) had household incomes greater than or equal to $100 000, and 202 of 209 participants (96.7%) were nonsmokers with self-reported very good or excellent health (154 of 208 participants [74.0%]).

**Table 1.** Baseline Characteristics of the Smart Cart Randomized Clinical Crossover Trial Participants^a^

Characteristic	Participants with data available, No.	Participants, No. (%)		
		Overall (N = 209)	Group 1 (AB) (n = 104)	Group 2 (BA) (n = 105)
Age, mean (SD), y^b^	209	55.4 (14.0)	53.2 (13.9)	57.5 (13.6)
Female	207	187 (90.3)	94 (91.3)	93 (89.4)
Non-Hispanic White	206	193 (94.1)	96 (93.2)	97 (94.2)
Bachelor’s degree or higher	208	102 (49.0)	50 (48.5)	52 (49.5)
Annual household income ≥$100 000	161	81 (50.3)	45 (52.3)	36 (48.0)
Nonsmoking	209	202 (96.7)	98 (94.2)	104 (99.1)
General health status	208			
Excellent or very good		154 (74.0)	79 (76.0)	75 (72.1)
Good		43 (21.0)	18 (17.3)	25 (24.0)
Fair or poor		11 (5.3)	7 (6.7)	4 (3.9)
Body mass index, mean (SD)^c^	198	25.5 (4.6)	25.2 (4.8)	25.7 (4.4)
Grocery Purchase Quality Index–16 score, mean (SD)				
Initial intervention period	209	41.1 (7.1)	41.2 (6.6)	41.0 (7.5)
Crossover intervention period	209	42.0 (7.3)	41.0 (6.8)	42.9 (7.7)
Spending on targeted foods, mean (SD), %				
Initial intervention period	209	32.0 (10.6)	32.0 (10.8)	31.0 (10.5)
Crossover intervention period	209	33.0 (12.6)	32.0 (13.1)	34.0 (12.1)
HEI-2020 score, mean (SD)^d^				
Baseline (n = 200)	200	72.7 (9.1)	73.0 (7.8)	72.5 (10.3)
Midpoint (n = 170)	170	71.5 (9.3)	70.8 (8.5)	72.3 (10.1)
Final (n = 170)	170	73.2 (9.0)	72.8 (7.9)	73.7 (10.1)

Abbreviation: HEI-2020, Healthy Eating Index–2010.

^a^ Group 1 received the treatment in period 1 followed by an active control in period 2 (AB design). Group 2 received the active control in period 1 followed by the intervention in period 2 (BA design).

^b^ After excluding participants whose spending was outside typical spending during the study period to create the analytic sample, age significantly differed at baseline between group 1 and group 2 (*P* = .02).

^c^ Body mass index is calculated as weight in kilograms divided by height in meters squared.

^d^ The HEI-2010 scores were a secondary outcome with a different analytic sample. Participants who reported eating less than 1000 kcal or more than 4500 kcal were deemed implausible^30^ and removed (24 participants at baseline, 23 participants at washout, and 28 participants at study completion, with 54 unique participants deleted because of implausible values and 170 participants left before multiple imputation). Because there were no missing data at baseline, 200 participants were included to calculate baseline HEI-2010 scores. Multiple imputation was performed on HEI-2010 scores of 170 participants.

All 209 participants had GPQI-16 scores. During the initial intervention period, the mean (SD) GPQI-16 score was 41.1 (7.1), and a mean (SD) of 32.0% (10.6%) of spending was for targeted food groups. The mean (SD) baseline HEI-2010 score was 72.7 (9.1). Age differed between group 1 and group 2 in the analytical sample (mean [SD], 53.2 [13.9] vs 57.5 [13.6] years; difference, 4.31 years; 95% CI, −8.06 to −0.56; *P* = .02).

### GPQI-16 Scores and Percentage Spending on Targeted Foods

**Figure 2.**
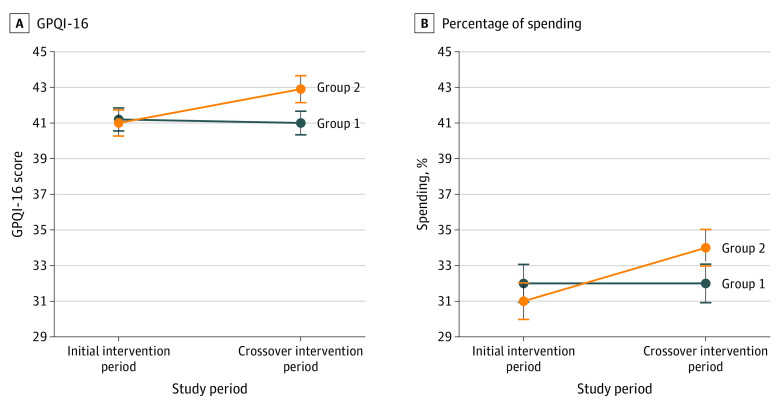
Grocery Purchase Quality Index (GPQI)–16 Scores and Percentage Spending on Targeted Food Groups Among 209 Smart Cart Study Participants Data points are means, with SEs denoted with error bars. Group 1 (104 participants) was randomized to receive personalized coupons in the initial intervention period followed by an active control in the crossover intervention period. Group 2 (105 participants) was randomized to receive the active control in the initial intervention period followed by personalized coupons in the crossover intervention period.

Figure 2 shows the changes in GPQI-16 scores and percentage spending on targeted foods in the initial intervention and crossover intervention periods. For both outcomes, there were no significant carryover effects. The personalized coupon intervention was significantly associated with GPQI-16 scores during the initial intervention period (between-group difference, 1.06; 95% CI, 0.27-1.86; *P* = .01), and the effect size was larger during the crossover period (between-period difference, 0.89; 95% CI, 0.09-1.69; *P* = .03). During the initial intervention period, participants in group 1 who received the personalized coupon intervention had marginally higher GPQI-16 scores than participants in group 2 (mean [SD], 41.2 [6.6] [95% CI, 39.9-42.5] vs 41.0 [7.5] [95% CI, 39.5-42.4]). The effect size was larger in the crossover period, and participants in group 2 who were exposed to the intervention had GPQI-16 scores 1.9 points higher than group 1 participants (mean [SD], 42.9 [7.7] [95% CI, 41.4-44.4] vs 41.0 [6.8] [95% CI, 39.7-42.3]), representing a 4.6% between-group difference in purchase quality. The personalized coupon intervention was also associated with percentage spending on targeted foods during both the initial intervention period (between-group difference, 1.38%; 95% CI, 0.08%-2.69%; *P* = .04) and the crossover period (between-period difference, 1.48%; 95% CI, 0.18%-2.78%; *P* = .03). During the initial intervention period, group 1 participants spent 1% more than group 2 participants on targeted healthy foods (mean [SD], 32.0% [10.8%] [95% CI, 30.1%-34.3%] vs 31.0% [10.5%] [95% CI, 29.4%-33.5%]). In the crossover period, group 2 participants spent 2.0% more on targeted foods than group 1 participants (mean [SD], 34.0% [12.1%] [95% CI, 32.0%-36.7%] vs 32.0% [13.1%] [95% CI, 29.8%-34.8%]).

The eFigure in Supplement 2 presents changes in the HEI-2010 scores in both the complete case analysis and after multiple imputation. Changes in HEI-2010 scores followed a pattern similar to that of the primary outcomes but were not statistically significant. There was a significant period effect observed (difference in HEI change score, 1.39; 95% CI, 0.20-2.58; *P* = .03 after multiple imputation), indicating that the intervention effect differed between study periods.

### Descriptive Changes in Food-Group Purchasing and Monthly Variability

Table 2 presents descriptive data without significance testing for the 11 GPQI-16 component scores and percentage spending within those categories. Components are organized in descending order according to the between-group difference in the crossover intervention period. Between-group differences were largest for total fruit, refined grains, whole fruit, and dairy. Purchasing changed the least for seafood and plant proteins, total protein foods, total vegetables, and whole grains.

**Table 2.** Grocery Purchase Quality Component Scores and Percentage Spending on Each Targeted Food Group

Variable	Mean (SD)			
	Initial intervention period		Crossover intervention period	
	Group 1 (AB)^a^	Group 2 (BA)^a^	Group (AB) 1^a^	Group 2 (BA)^a^
Total fruit				
Score	3.15 (1.32)	3.44 (1.34)	3.17 (1.38)	3.74 (1.39)
Percentage expenditure	2.0 (2.0)	2.0 (5.0)	1.0 (2.0)	2.0 (5.0)
Refined grains				
Score	0.79 (2.19)	0.92 (2.34)	0.97 (2.58)	1.47 (3.02)
Percentage expenditure	10.0 (10.0)	10.0 (10.0)	10.0 (10.0)	10.0 (10.0)
Whole fruit				
Score	3.09 (1.37)	3.39 (1.36)	3.26 (1.48)	3.69 (1.41)
Percentage expenditure	10.0 (6.0)	12.0 (7.0)	11.0 (7.0)	13.0 (7.0)
Dairy				
Score	7.89 (2.63)	8.16 (2.57)	7.70 (2.98)	7.88 (2.76)
Percentage expenditure	13.0 (6.0)	14.0 (7.0)	13.0 (7.0)	14.0 (10.0)
Greens and beans				
Score	2.02 (1.18)	2.00 (1.39)	2.04 (1.38)	2.15 (1.41)
Percentage expenditure	5.0 (3.0)	5.0 (3.0)	5.0 (3.0)	5.0 (3.00
Sweets				
Score	7.74 (2.23)	7.76 (2.23)	7.78 (2.28)	7.86 (2.39)
Percentage expenditure	5.0 (4.0)	4.0 (4.0)	5.0 (5.0)	4.0 (4.0)
Processed meat				
Score	4.24 (0.92)	4.23 (1.07)	4.26 (0.98)	4.30 (0.97)
Percentage expenditure	4.0 (4.0)	4.0 (6.0)	4.0 (4.0)	4.0 (4.0)
Whole grains				
Score	3.28 (2.69)	3.04 (2.80)	3.27 (2.58)	3.30 (2.75)
Percentage expenditure	4.0 (3.0)	3.0 (3.0)	4.0 (3.0)	4.0 (4.0)
Total vegetables				
Score	3.38 (1.09)	3.18 (1.34)	3.27 (1.28)	3.30 (1.37)
Percentage expenditure	14.0 (6.0)	13.0 (7.0)	13.0 (7.0)	13.0 (6.0)
Total protein foods				
Score	3.06 (1.48)	2.68 (1.51)	2.92 (1.48)	2.93 (1.55)
Percentage expenditure	8.0 (7.0)	7.0 (6.0)	8.0 (8.0)	8.0 (8.0)
Seafood and plant protein				
Score	2.51 (1.89)	2.16 (1.80)	2.31 (1.78)	2.25 (1.77)
Percentage expenditure	6.0 (5.0)	5.0 (6.0)	6.0 (9.0)	5.0 (5.0)

^a^ Group 1 (AB) (n = 104) was randomized to receive personalized coupons in the initial intervention period followed by an active control in the crossover period. Group 2 (BA) (n = 105) was randomized to receive the active control in the initial intervention period followed by personalized coupons in the crossover period.

**Figure 3.**
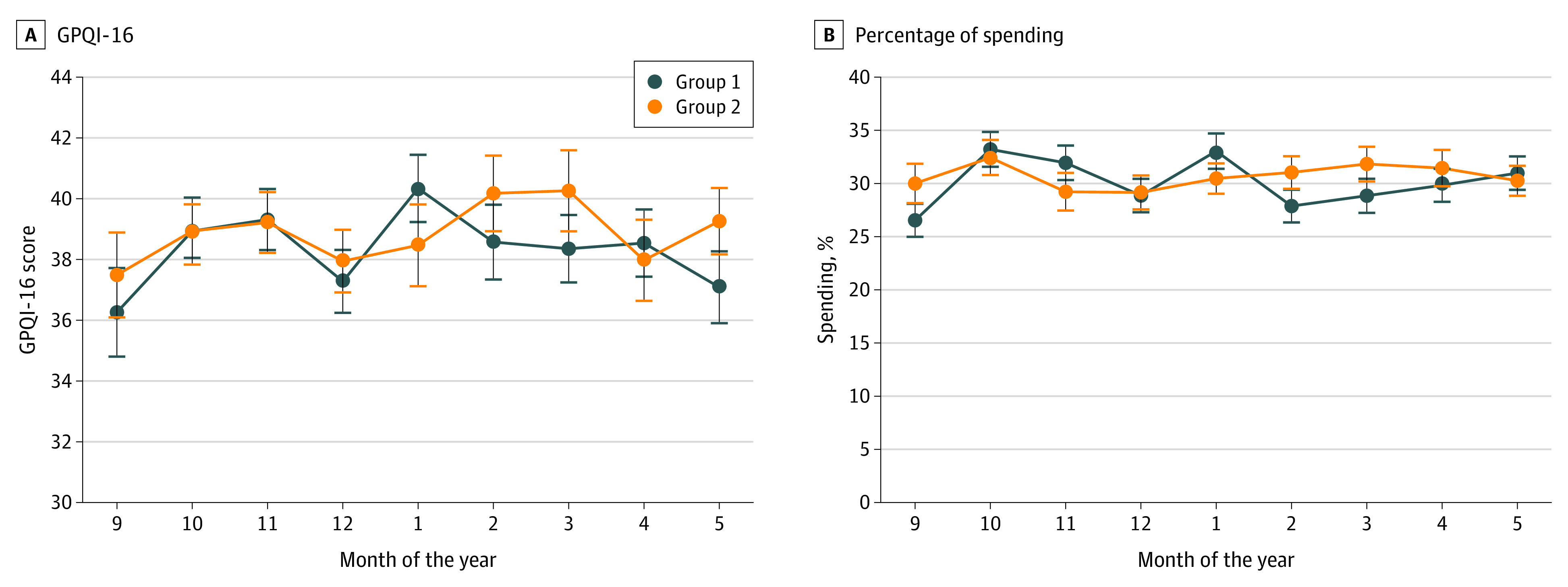
Monthly Grocery Purchase Quality Index (GPQI)–16 Scores and Percentage Spending on Targeted Food Groups Data points are means, with SEs denoted with error bars. Group 1 (104 participants) was randomized to receive personalized coupons in the initial intervention period followed by an active control in the crossover period. Group 2 (105 participants) was randomized to receive the active control in the initial intervention period followed by personalized coupons in the crossover period. The x-axis (months) shows the study duration from September 2018 through May 2019; the end of the initial intervention period occurred in December 2018, and the beginning of the crossover intervention period occurred in February 2019.

Figure 3 presents descriptive data without significance testing on participants’ monthly GPQI-16 scores and percentage spending on targeted foods. Group 1 participants (AB sequence) had the greatest increase in percentage spending on targeted foods during the first month of the study; subsequently, spending on targeted foods tapered and stabilized. Group 2 participants generally had lower monthly spending on targeted items than group 1 participants during the initial intervention and washout periods, but percentage spending on targeted items increased and exceeded group 1’s spending during the crossover intervention period.

### Process Measures

The coupon format was similar across study periods, and 76% of personalized coupons were for a percentage off the list price. Coupon redemption rates for participants receiving personalized coupons were 7% in the first period and 10% in the second period; participants opened emails a mean (SD) of 2.28 (2.53) times per week and shopped a mean (SD) of 1.61 (1.01) times per week (eTable 1 and eTable 2 in Supplement 2).

## Discussion

To our knowledge, this 9-month pilot randomized clinical crossover trial is the first to use detailed individual-level information to develop a semiautomated, personalized healthy dietary incentives platform to improve food purchasing behavior and overall diet quality. Personalized healthy food incentives modestly improved both primary study outcomes, with more meaningful changes observed in the crossover intervention period. These results demonstrate that the intervention led to a small but significant improvement in grocery purchase quality through increased purchase of foods targeted by the intervention. Furthermore, participants’ descriptively healthier dairy and refined grains purchases indicate that categories beyond produce are responsive to price changes.

Most dietary interventions are resource intensive, requiring individual counseling, and are associated with modest, short-term effects for a small population.^39,40,41,42^ Prior grocery purchase interventions have increased produce intake between 0.24 cups (an approximately 1-point increase in HEI-2010)^43^ and 1.5 cups per day.^22^ Our pilot study demonstrated that semiautomated, data-driven personalized dietary incentives adapted to individual preferences improved overall dietary patterns, with an effect size similar to those of prior studies. Small and sustained improvements in dietary intake, when extrapolated to the population level, can have long-term effects on health care spending, morbidity, and mortality.^44,45^ Importantly, the semiautomated Smart Cart platform provides a foundation for developing a fully automated machine-learning platform that would reduce resources needed for intervention delivery and improve efficacy by refining personalization. In the future, these types of interventions could challenge existing retailer-driven marketing strategies focused solely on profit^46^ and influence population-level dietary patterns.

Purchase of incentivized foods appeared driven by the personalized suggestions more than the coupons per se in the present study. Although redemption rates were higher than the national 1% average,^47^ they ranged from 7% to 10%, whereas email open rates were more than 2 times per week. The observed improvement in primary study outcomes despite limited coupon use suggests that personalization may cost-effectively improve purchase quality. It also supports previous research suggesting that mere exposure to coupons may be independently associated with purchasing behavior.^48^

Nonetheless, changing certain aspects of intervention delivery may improve coupon redemption and purchase quality. In the future, using a mobile telephone application could enhance coupon accessibility.^49^ Participants also needed to scan their loyalty card at the beginning of the transaction for coupons to apply. Some research suggests that the regularity that coupons are sent can be negatively associated with redemption^48^; sending coupons at irregular intervals may have enhanced overall redemption, as would sending coupons with shorter expiration lengths^50^ while participants were physically in the store.^51^ Coupon format was also not considered and requires evaluation in future studies.

The modest association of the present study with increasing healthy food purchases is consistent with the personalized dietary incentive trial in New Zealand^12^ and contrasts with the trial in South Africa.^25^ Barriers to coupon use identified in previous trials included the burden of sorting through lists to identify healthier foods^12^ and insufficient understanding of how the type, mode, and frequency of personalized feedback were associated with use.^25^Although the Smart Cart study limited the number of options delivered to participants simultaneously, seamlessly integrating incentive-based interventions with the shopping experience is necessary.

Further development of personalized healthy food incentives aligned with individuals’ multidimensional preferences is warranted.^52^ Currently, marketing firms leverage available consumer data to anticipate consumers’ evolving preferences,^53^ and within health care, recommender systems have become essential for evaluating drivers of patient behavior.^54^ Implementing such systems to improve purchasing behavior is a natural extension if customer privacy is protected and predatory marketing tactics that perpetuate health disparities are not applied.^55^

### Limitations

The findings of the present pilot study should be interpreted in the context of some limitations. There were some technical challenges working with the register system initially that may have reduced the intervention’s effectiveness. Our inclusion criteria may not have eliminated all seasonal residents who depart during the winter. The study sample was affluent and educated and had high baseline diet quality, with mean HEI-2010 scores approximately 14 points higher than the US population.^56^ Thus, these results are not generalizable to US adults and likely are attenuated from what would be expected with heterogenous samples,^43^ especially among racial/ethnic and socioeconomic groups where disparities in diet quality have been observed.^57,58,59^ Furthermore, the systems used to classify UPC codes and select healthy food coupons require refinement to reduce manual input required from nutrition experts.

## Conclusions

Results from this novel, pilot, randomized, clinical crossover trial provide promising evidence to advance machine-learning algorithms to disseminate personalized, healthy food incentives in diverse populations to improve diet quality. Although the findings demonstrated a modest effect size, the study used an innovative design that aligned academic and retailer interests, which is an essential component for scaling this work in the future. Proof-of-concept that personalized healthy food incentives improve the quality of grocery purchases even among participants with high diet quality highlights the potential impact of expanding this tool to underserved groups. Thus, the potential for data analytics to enhance dietary interventions is compelling as public health practitioners continue developing multipronged strategies and partnerships with retailers and health insurers to facilitate behavior change.

## Notes

Supplement 1. **Trial Protocol**

Supplement 2. **eFigure.** Change in Healthy Eating Index 2010 Scores Among 170 Smart Cart Study Participants With Valid Dietary Data **eTable 1.** Coupon Format Frequency in the Smart Cart Study **eTable 2.** Email Open Rate and Mean Shopping Trips per Week in the Smart Cart Study

Supplement 3. **Data Sharing Statement**
