# Supplementary material for: Effect of Personalized Incentives on Dietary Quality of Groceries Purchased: A Randomized Crossover Trial
Source: JAMA Netw Open. 2021 Feb 10;4(2):e2030921. doi: 10.1001/jamanetworkopen.2020.30921 (PMC7876589; doi:10.1001/jamanetworkopen.2020.30921)
Supplement: Supplement 1. — Trial Protocol [file jamanetwopen-e2030921-s001.pdf]

1 Supplement 1 A Trial Protocol

THE UNIVERSITY OF RHODE ISLAND
DIVISION OF RESEARCH AND ECONOMIC DEVELOPMENT

IRB
Initial IRB Application
Rev. March 2015

OFFICE OF RESEARCH INTEGRITY
HUMAN SUBJECTS RESEARCH

The University of Rhode Island **(URI) Institutional Review Board** (IRB) is under the governance of the Office of Human Subject Research Protection. The **Office of Human Subject Research Protection** (OHRP) is the federal agency under the Department of Health and Human Services (DHHS) responsible for implementing regulations (45 CFR 46) governing Biomedical, Behavioral and Social Sciences research involving human subjects. DHHS requires federally funded research to be monitored for compliance with its regulations by an institutional Human Subject Research Protection Program. URI fosters a culture of compliance and requires all research involving humans, regardless of sponsorship, to comply with the regulations governing human subject research.

The protective oversight for human subject research activities are delegated to the IRB to assure compliance with the governing federal regulations for human subject research set forth by OHRP.

The IRB is charged with assuring that all human subject research, regardless of sponsorship, conducted at the URI complies with the federal regulations (45 CFR 46) of the Department of Health and Human Services (DHHS), Food and Drug Administration (FDA), the Belmont Report, State and Local Laws, and IRB policies and procedures.

All human subject research must be reviewed, prospectively approved, and subject to continuing oversight (at least annually) by the IRB to assure the safety and welfare of research participants remains in compliance with governing federal regulations and guidance.

Research requiring Registration

Use this form to register research involving human subjects.
Human subject use may not proceed until authorization from the IRB is received.

Form Submittal

Submit via IRBnet the following:

- This IRB Application. **Please try to limit your answers to the space provided.** Upload separate document if you believe additional information is valuable to the committee (refer to specific questions you are addressing).
- Any attachments **(e.g., appendices, consent form(s), assent form(s), flyers, etc.)**
- CITI certification **(Human Subjects Group 1 (Social Behavioral))** for all key personnel (no more than 3 years old)
- Relevant thesis, dissertation, or grant proposals
- Signed Proposal Approval Signature Sheet, if part of a thesis/dissertation MA/PhD
- Student Assurance form, if graduate student will be using project data to complete a thesis/dissertation

Adobe Forms
- **Check that you have installed the latest version of Adobe Acrobat or Reader. The link to install Adobe Reader is: http://get.adobe.com/reader.**

- Save the form once you have entered your information.

To Download the Protocol for Mac and iOS Users - open the file using Adobe Reader rather than the Preview function built into your Mac OS. ***For Windows users*** - open the file using Adobe Acrobat or Reader rather than using a web browser.

Training

All faculty, staff, and students listed on the Protocol must complete the on-line training course at www.CITIprogram.org. Register as a new user and choose URI as your institution. Complete the **Human Subjects Group 1 (Social Behavioral)** course. The IRB Administrator will alert you if other training modules are also required.

Questions?

- Contact the Office of Research Integrity at 401-874-4328 or email: researchintegrity@etal.uri.edu
- For training materials on IRBNet or the Human Subject Research Policy, refer to the Office of Research Integrity website.

IRB
Initial IRB Application
Rev. March 2015

Add all documents checked in this list to IRBNet Package

Application Contents

Indicate the documents being submitted for this research project. Check all appropriate boxes.

- ☒ **IRBNet Package Summary Form - REQUIRED FOR ALL IRBNet SUBMISSION PACKAGES**
- ☒ **Initial IRB Application of Human Subjects Research - REQUIRED**
- ☒ **CITI certification for all personnel - REQUIRED**
- ☒ Consent form(s), Assent Form(s), Permission Form(s), and Verbal Script(s), including translated documents
- ☐ Data Collection Form(s) for Investigator-Initiated Studies **(Section 11, pg. 10)**
- ☐ Data Collection Form(s) involving protected health information (**Appendix N**)
- ☒ Recruitment Materials (e.g., ads, flyers, telephone or other oral script, radio/TV scripts, internet solicitations)
- ☐ Script(s) or Information Sheet(s), including Debriefing Materials
- ☐ Instruments (e.g., questionnaires or surveys to be completed by participants) **(Section 11, pg.10)**
- ☐ Other Committee Approvals/Letters of Support
- ☐ HIPAA Research Authorization Form(s)
- ☒ Research Protocol or Proposal **- REQUIRED FOR MA/PHD STUDENTS**
- ☐ MA/PhD Proposal Approval Form **- REQUIRED FOR MA/PHD STUDENTS**
- ☒ Complete Grant Application or Funding Proposal, as applicable
- ☐ Drug Manufacturer's Approved Labeling/Investigator's Drug Brochure (**Appendix F**)
- ☐ Device Manufacturer's Approved Labeling (**Appendix E**)
- ☐ Other supporting documentation and/or materials

Appendices

If box is checked, appendix must be included in IRBNet submission package for processing.

- ☐ **Appendix A:** Exempt Review **(See section 1, pg. 3)**
- ☒ **Appendix B:** Expedited Review - Initial Review **(See section 1, pg. 3)**
- ☐ **Appendix C:** Data Repositories **(See section 11, pg.10)** Definition --> Retain data for future research
- ☐ **Appendix D:** Deception **(See section 11, pg.10)**
- ☐ **Appendix E:** Devices **(See section 11, pg.10)**
- ☐ **Appendix F:** Drugs or Biologics **(See section 11, pg.10)**
- ☐ **Appendix G:** Genetic Testing **(See section 11, pg.10)**
- ☐ **Appendix H:** Storage of Biological Materials **(See section 11, pg.10)**
- ☐ **Appendix I:** Minors **(See section 14, pg. 12)**
- ☐ **Appendix J:** Non-English Speaking Participants **(See section 14, pg. 12 and section 18, pg.14)**
- ☐ **Appendix K:** Pregnant Women/Fetuses/Neonates **(See section 14, pg. 12)**
- ☐ **Appendix L:** Prisoners **(See section 14, pg. 12)**
- ☐ **Appendix M1:** Waiver or Alteration of Consent Process **(See section 11, pg. 10 and section 18, pg. 14)**
- ☐ **Appendix M2:** Waiver of Signed Consent **(See section 18, pg.14)**
- ☐ **Appendix N:** Waiver or Alteration of HIPAA Research Authorization **(See section 21, pg. 17)**
- ☐ **Appendix O:** Research in International Settings **(See section 7, pg. 6)**
- ☐ **Appendix P:** Radiation **(See section 11, pg. 10)**
- ☐ **Appendix Q:** Adults with Decisional Impairment **(See section 14, pg.12)**
- ☐ **Appendix R:** Change in Personnel **(See section 2, pg. 3)**
- ☐ **Appendix V:** Unaffiliated Investigator Agreement
- ☐ **Appendix X** - Conflict of Interest in HSR

IRB
Initial IRB Application
Rev. March 2015

REMINDER FOR MAC USERS:
Complete form in Adobe Reader, not the Preview function in MAC OS. Using the Preview function will disable parts of the form.

Section 1 - Administration

Project Title: Individually-targeted incentives, diet quality, and health outcomes among adults

Create Project Short Title: Individually-targeted incentives study

Research Start Date: **UPON IRB APPROVAL**

I would like to defer the start date of my research to: (may be no more than 1 year)

Anticipated Research End Date: 7/17/2020

Select level of review that applies to this project: ☐ **Full** ☐ **Exempt --> Complete Appendix A** ☒ **Expedited --> Complete Appendix B**

Section 2 - Personnel

List all personnel associated with the project

Principal Investigator (PI)

Principal Investigator MUST be URI Faculty or Staff

Name	Maya Vadiveloo	College	Health Services
Email	maya_vadiveloo@uri.edu	Department	Nutrition and Food Sciences
Position	Faculty	Phone Number	401-874-2992

If Non-URI, check box ☐ FWA # and Institution

If Non-URI, submit Appendix V - Unaffiliated Investigator Agreement in IRBNet Package.

Check all responsibilities that apply: ☒ Consent Subject ☒ Recruit ☒ Protocol Design ☒ Data Analysis ☒ Intervention

Co-Investigator

Name	Stephen Atlas	College	Business Admin
Email	satlas@uri.edu	Department	Marketing
Position	Faculty	Phone Number	401-874-4190

If Non-URI, check box ☐ FWA # and Institution

If Non-URI, submit Appendix V - Unaffiliated Investigator Agreement in IRBNet Package.

Check all responsibilities that apply: ☒ Consent Subject ☒ Recruit ☒ Protocol Design ☒ Data Analysis ☒ Intervention

Co-Investigator

Name	Ashley Buchanan	College	Pharmacy
Email	Buchanan@uri.edu	Department	Pharmacy Practice
Position	Faculty	Phone Number	401-874-4739
If Non-URI, check box ☐		FWA # and Institution	

If Non-URI, submit Appendix V - Unaffiliated Investigator Agreement in IRBNet Package.

Check all responsibilities that apply: ☒ Consent Subject ☒ Recruit ☒ Protocol Design ☒ Data Analysis ☒ Intervention

Co-Investigator

Name		College	
Email		Department	
Position		Phone Number	
If Non-URI, check box ☐		FWA # and Institution	

If Non-URI, submit Appendix V - Unaffiliated Investigator Agreement in IRBNet Package.

Check all responsibilities that apply: ☐ Consent Subject ☐ Recruit ☐ Protocol Design ☐ Data Analysis ☐ Intervention

MA/PhD Researcher

Name		College	
Email		Department	
Position		Phone Number	
If Non-URI, check box ☐		FWA # and Institution	

If Non-URI, submit Appendix V - Unaffiliated Investigator Agreement in IRBNet Package.

Check all responsibilities that apply: ☐ Consent Subject ☐ Recruit ☐ Protocol Design ☐ Data Analysis ☐ Intervention

Will this project be used as a thesis or dissertation proposal? ○ Yes ○ No

If YES, please submit:
- Signed MA or PhD Proposal Approval Form
- Proposal Document

Other Key Personnel

Name	Position	Non-URI?	Responsibilities: Consent Subject	Recruit	Protocol Design	Data Analysis	Intervention
Xintong Guan	Graduate Student	☐	☑	☑	☐	☑	☑
		☐	☐	☐	☐	☐	☐
		☐	☐	☐	☐	☐	☐
		☐	☐	☐	☐	☐	☐
		☐	☐	☐	☐	☐	☐

Key personnel are defined as individuals who participate in the design, conduct, or reporting of human subjects research. At a minimum, include individuals who recruit participants, obtain consent, or who collect study data.

Are there multiple PIs at different sites?
○ Yes
◉ No
If **Yes** --> answer questions in **Section 7c**.

Is the URI PI the lead investigator?
◉ Yes
○ No
If **No** --> PI Name:
PI Institution:
Is an agreement with the other institution requested?
○ Yes
○ No

Section 3 - Education

Educational requirements (initial and continuing) must be satisfied prior to submitting the application for IRB review. See Human Subjects Protection Training or contact ORI for more information.

Have all University of Rhode Island investigators and key personnel completed the required web-based course (CITI) in the protection of **Human Research Subjects (Basic Course)?**
◉ Yes
○ No

Attach PDF of CITI Training Certificate for all personnel listed

Section 4 - Financial Conflict of Interest (FCOI)

A **financial conflict of interest** may exist whenever financial considerations or publication rights have the potential to compromise or have the appearance of compromising one's professional judgment and independence in the design, conduct or publication of research.

The IRB considers the investigator's financial interests when evaluating the protection of human subjects. If a financial interest is reported, the IRB will assess the investigator's objectivity in:

- Communicating Risks
- Selecting Subjects
- Promoting informed consent
- Gathering, analyzing, and reporting data

Does any University of Rhode Island investigator (including principal or co-investigator), key personnel, or their immediate family members have a financial interest (including salary or other payments for services, equity interests, or intellectual property rights) that would reasonably appear to be affected by the research, or a financial interest in any entity whose financial interest would reasonably appear to be affected by the research?
○ Yes --> Complete **Appendix X - Conflict of Interest in Human Subjects Research**
◉ No

Section 5 - Funding or Other Support

If the research is federally funded and involves a subcontract to or from another entity, an IRB Authorization Agreement may be required. Contact ORI for more information.

a. Is the research funded or has funding been requested? ◉ Yes ◯ No

If **Yes** --> Specify funding agency and support: Foundation for Food and Agriculture Research

☒ ***By checking this box, you have provided a copy of the grant application or funding proposal in your IRBNet submission package. The university is required to verify that all funding proposals and grants (new or renewals) have been reviewed by the IRB before funds are awarded.***

b. Is any support other than monetary (e.g., drugs, equipment, etc.) being provided for the study? ◯ Yes ◉ No

If **Yes** --> Specify provider or agency and support:

Section 6 - Other Institutional Approvals

Check all that apply and provide applicable documentation. ***IRB review cannot be conducted until required institutional approvals or exemptions are obtained, except as noted.***

☐ **Institutional Biosafety Committee (IBC)** - Approval required for research activity involving:
- Recombinant DNA (rDNA)
- Biological agents (i.e., viable infectious microorganisms (including prions) regardless of their pathogenicity to humans)
- Human or nonhuman primate materials **(e.g., blood, unfixed tissue, cell lines, finger sticks and blood draws)**
- Biological toxins subject to the National Select Agents Registry Program managed by the U.S. Departments of Health and Human Services (HHS) and Agriculture (USDA).

☐ **Conflict of Interest Management Committee (CIMC)** - CIMC is responsible for the review and assessment of all financial disclosures related to research projects at URI and for determining any actions required to ensure that real or perceived financial conflicts of interest are managed or eliminated.

☒ None

Section 7 - Location of Research

a. List the URI specific site(s) at which the research will be conducted

Location Name (or description)	Address
Fogarty Hall	41 Lower college road

Research to be conducted outside of URI facilities will minimally require a LETTER OF AUTHORIZATION (on institutional letterhead) and may require another IRB's approval if personnel are engaged. See OHRP Engagement Guidance or contact ORI for more information.

b. Location of Research?

☒ Domestic Sites --> ***Upload a letter of authorization, on institution letterhead, as applicable***

☐ International Sites --> ***Upload a letter of authorization, on institutional letterhead and*** ***Complete Appendix O***

c. List the **non-URI** specific site(s).

Location Name (or description)	Address
Belmont market	600 Kingstown Rd, Wakefield, RI 02879

Has a letter of authorization for each non-URI site been uploaded to IRBNet? ◉ **Yes** ○ **No**

d. Are there multiple PIs at different sites? ○ Yes ◉ No

If **Yes**, complete the following:

ii. Is an insitutional IRB agreement (IAA) requested or in process for this protocol? ○ Yes ○ No

If Yes, please elaborate.

ii. Describe the communication between sites that might be relevant to the protection of participants, such as unanticipated problems, interim results, and protocol modifications.

iii. Describe IRB oversight arrangements for each collaborative site (i.e., who will provide IRB review and approval). ***Upload copies of the non-URI approvals, as applicable. Contact ORI if requesting that the University of Rhode Island serves as the IRB of record.***

Have IRB approvals been included? ○ **Yes** ○ **No**

Section 8 - Summary of Research

Summarize the proposed research using ***non-technical*** language that can be readily understood by someone outside the discipline. Explain briefly the:

- **research design**
- **procedures to be used**
- **risks and anticipated benefits**
- **the importance of the knowledge that may reasonably be expected to result.**

Use complete sentences.

This project proposes to evaluate the impact of individually-tailored coupons to motivate a sample of adults to purchase foods aligned with healthier dietary patterns shown to improve overall health. The goal of this study is to test whether targeting coupons will improve diet quality and health parameters more than a "one size fits all" approach toward healthy coupon incentives.

We propose to examine food purchase history from existing scanner data along with other individual-level sociodemographic and health data to develop targeted coupons that shift consumer purchasing patterns from less healthful dietary purchases to healthier substitutes.

In order to test the effectiveness of targeted vs. untargeted incentives on the dietary quality of food purchases, dietary intake, and health metrics, we propose using a cross-over design with block randomization to ensure balance in the size of the two study arms. In period 1, participants will be randomized to the treatment or control condition using a randomization scheme provided by the team Biostatistician. Those randomized to the treatment group will receive targeted weekly coupons for healthier substitutions based on their purchase history and other individual-level metrics. Those randomized to the control group will receive 5% discount on their purchases and occasionally untargeted coupons. Both groups will receive general information notification. After a 3-month period, there will be a 2-month washout period when no coupons are sent to either group. In period 2, we will follow the participants for a 3-month period where each group receives the study intervention they did not receive in period 1. During each 3-month treatment period, participants in the treatment group will receive a set of weekly coupons that can be used within 2 weeks. These coupons and the delivery mechanism will be developed through the food environmental assessment. Following participant enrollment, trained research assistants will collect information about each participant's usual diet, using iPads and Qualtrics survey software for both intervention and control groups at baseline, 3 months, 6 months, and 9 months. This study will involve a focus group to learn about shopper's purchasing habits and preferences and investigate factors that influence people's shopping behaviors and coupon using behaviors.

For all types of data, the risk of participating in this study is minimal. If participants are uncomfortable with any of the very standard and routine questions that are asked, they have the ability to opt out of responding and continue with the study. All participants will benefit by receiving 5% of their grocery purchases and up to $10 in coupons each week. Additionally this project will provide insights about developing an intervention that motivates shoppers with varied dietary patterns toward making healthier food purchases, using loyalty card data.

Section 9 - Scientific Background & Literature Review

Summarize existing knowledge and previous work that support the expectation of obtaining useful results without undue risk to human subjects. ***Use complete sentences.***

Most Americans have poor dietary behaviors but dietary patterns are difficult to change and difficult to target. Financial incentives are increasingly being applied to promote healthier dietary patterns. In a recent randomized controlled trial within a New York City supermarket, a 50% discount on fruit and vegetables led to a 300% increase in fruit and vegetable purchases and doubled fruit and vegetable intake. Yet, questions remain about the feasibility of implementing cost-effective and sustainable healthy eating incentive programs. Empirical research in marketing finds that customized incentives are more effective at influencing customers' current and future purchase behavior than a standardized "one size fits all" approach. For example, when CVS's loyalty program used customer purchase history to promote products for the first time, CVS's total sales increased 10%. However, health researchers have not applied this foundational marketing principle toward promoting healthful food purchases and they have also found that incentive targeting effectiveness is constrained by knowledge about individual-level differences in responsiveness to various incentives.

Therefore, our research is going to address both these gaps by examining whether tailored incentives increases the purchase of healthy foods and whether other individual-level data gathered from loyalty cards and participant surveys can be used to more efficiently allocate financial incentives for improving dietary quality and health.

Section 10 - Research Objectives

List the specific scientific or scholarly aims of the research study. ***Use complete sentences.***

This project is going to:

Aim 1: Conduct an environmental assessment of the food landscape at a selected retail location (Belmont market) and identify healthier substitutes in the store environment to develop an assortment of targeted coupons.

Aim 2: Examine the effect of targeting coupons using past purchasing history and other individual sociodemographic and dietary information on the likelihood of purchasing the healthier targeted food and the resulting quality of all foods purchased and consumed.

Aim 3: Examine the short-term relationship between food choice, dietary quality, and health outcomes (i.e. body mass index and self-reported health status), and explore which groups of consumers are most responsive to targeted health incentives.

Section 11 - Research Methods & Activities

a. Identify and describe all interventions and interactions that are to be performed solely for the research study. Distinguish research (i.e., experimental) activities from non-research activities. ***Provide description (e.g., spreadsheet, forms, or flow charts) of data being collected. Do not include case report forms for multi-site industry-sponsored or cooperative group studies.***

We propose to enroll a convenience sample of customers to conduct a cross-over randomized trial to examine how targeting healthful coupons based on purchase history influences food purchases.

We will track purchase habits using a 3rd party secure data company (Accelitec). At baseline, we may conduct brief interviews with shoppers about their shopping habits, diet preferences and preferences for receiving coupons to help inform which foods to incentivize and how to deliver coupons to them. Research assistants also observe shoppers in the store to detect high traffic areas.

In period 1, participants will complete an informed consent, and either enroll in Belmont's loyalty program, or provide researchers loyalty card information. Participants will be randomized after completing baseline information collected on iPads using Qualtrics including: 1. Sociodemographic information; 2. Dietary Measures: Viocare, a validated an online graphical food frequency questionnaire, the result of which will be used to compute the Healthy Eating Index; 3.Health parameters, such as health condition and health behaviors; 4. Dietary Preferences such as preference for organic foods. Examples of the types of questions are attached. The treatment group will then receive targeted weekly coupons for healthier substitutions based on their purchase history and other individual-level metrics for 3-months. The control group will receive a 5% discount and occasional coupons irrespective of their purchase history. After a 3-month period, there will be a 2-month washout period when no coupons are sent to either group. During the washout period, above questions will be asked again for each group.

In period 2, we will then follow the participants for another 3-month period where each group receives the study intervention they did not receive in period 1. Similar questions will be asked again for each group after the 3-month treatment period.

This study will involve a focus group to learn about shopper's purchasing habits and preferences and investigate factors that influence people’s grocery shopping behaviors and coupon using behaviors.

Throughout and study, all participants will receive weekly, biweekly or monthly emails or text messages with meal ideas and recipes.

We will use block randomization technique to reduce bias and achieve balance in the allocation of participants.

Statistical Analysis Plan:

First, we will examine the purchase rate data (percent of total items purchased that are couponed items) and Grocery Purchase Quality Index-2016 scores for normality graphically (i.e. histograms and Q-Q plots), identifying and removing any outliers present. We will examine the change in purchase rate and healthfulness using either a t-test or, if the data is not normal, a Wilcoxon Rank sum tests at the end of the full 7-month crossover study using a two-sided test with a 5% Type I error rate.

b. Check all research activities that apply:

**General Intervention (Social and Medical)**	
☐ Data repositories --> Complete **Appendix C** (future unspecified use, including research databases)	☐ Program Protocol (Umbrella Protocol)
☒ Randomization	Other --> Specify:

**Bio/Medical Intervention**	
☐ Anesthesia (general or local) or sedation	☐ Non-invasive medical procedures (e.g., EKG, Doppler)
☐ Biohazards (e.g., rDNA, infectious agents, select agents, toxins) - **IBC Approval Required**	☐ Placebo
☐ Biological sampling (other than blood) - **IBC Approval Required**	☐ Pregnancy Testing
☐ Blood drawing - **IBC Approval Required**	☐ Radiation (e.g., CT or DEXA scans, X-rays, nuclear medicine procedures) --> Complete **Appendix P**
☐ Devices --> Complete **Appendix E**	☐ Record Review - **If HIPAA, include Appendix N**
☐ Drugs or biologics --> Complete **Appendix F**	☐ Specimen Research
☐ Food Supplements	☐ Stem Cell Research - **IBC Approval Required**
☐ Gene Transfer	☐ Storage of Biological Materials --> Complete **Appendix H & C** (future unspecified use, including repositories)
☐ Genetic Testing --> Complete **Appendix G**	☐ Surgical Procedures (including biopsies or other invasive medical procedures)
☐ Magnetic Resonance Imaging (MRI)	Other --> Specify:

**Social Intervention**	
☐ Audio, video, digital, or image recordings	☐ Data, publicly available (**Provide source below**)
☐ Deception --> Complete **Appendix D & M1**	Provide source:
☐ Diet, exercise, or sleep modifications	☒ Data, not publicly available (**Provide source below**)
☒ Focus Groups	Provide source: Accelitec
☐ Internet or e-mail collection	☒ Surveys, questionnaires, or interviews (one-on-one)
☐ Materials that may be considered sensitive, offensive, threatening or degrading	☐ Surveys, questionnaires, or interviews (group)
☐ Oral history (does not include medical history)	☒ Observation of participants (including field notes)
	Other --> Specify:

Section 12 - Duration

Informed consent must state total time required

Estimate the time required from each participant, including individual interactions, total time commitment, and long-term follow-up, if any. **(If complicated design, please attach a flowchart)**.

The whole study will last for 9 months, including 3-month treatment period after the coupon distribution, 2-month washout period when no coupons are sent to either group, 3-month follow up period where each group receives the study intervention they did not receive in period 1.

At the beginning of the study, during the washout period and after the follow up period, participants will be asked to respond to short surveys using qualtrics The interviews will take 45 minutes to 1 hour, in total 1-2.5 hours.

Section 13 - Number of Participants

The number of participants is defined as the number of individuals who agree to participate (i.e., those who provide consent or whose records are accessed, etc.) even if all do not prove eligible or complete the study. The total number of research participants may be increased only with prior IRB approval.

a. Provide the total number of participants (or number of participant records, specimens, etc.) for whom you are seeking URI IRB approval.

225

b. Explain how this number was derived (e.g., statistical rationale, attrition rate, etc.).

Recruiting a 225 participant sample size account for incomplete or illegible responses. A final sample size approximately 200, 100 for each group is needed to detect a meaningful difference between treatment group and control group and a desired small to medium effect size that could explain a meaningful improvement in dietary quality, developed in consultation with Dr. Ashley Buchanan, a biostatistician on the project.

c. Is this a multi-site study?

○ Yes --> Indicate total number of participants to be enrolled across all sites, including multiple PIs at different sites:

◉ No

Section 14 - Participation Population

a. Specify the age ranges(s) of the individuals who may participate in the research:

Age Range(s): 18 years or older

b. Specify the participant population(s). Check all that apply:

☒ Adults	☐ Pregnant women/fetuses --> Complete **Appendix K**
☐ Children (<18 years) --> Complete **Appendix I**	☐ Neonates (uncertain viability/nonviable --> Complete **Appendix K**
☐ Adults with decisional impairment --> Complete **Appendix Q**	☐ Prisoners --> Complete **Appendix L**
☐ Non-English speaking --> Complete **Appendix J**	☐ Unknown (e.g., secondary use of data/specimens, non-targeted surveys, program protocols)
☐ URI student research pools (e.g., psychology, linguistics, non-targeted surveys, program protocols) **-->**	Specify:

c. Describe the characteristics of the proposed participants, and explain how the nature of the research requires/justifies their inclusion.

Participants must be 18 years or older, speak English, not Belmont employees or pregnant, and report purchasing at least half of their weekly groceries at Belmont market.

d. Will any participants be excluded based on age, gender, race/ethnicity, pregnancy status, language, education, or financial status?

◉ Yes
○ No

If **Yes** --> Explain the criteria and reason(s) for each exclusion. ***Consider the study's scientific or scholarly aims and risks.***

Belmont market employees will be excluded from the study. People don't speak English, purchase less than half of the groceries in Belmont, and women who are pregnant or plan to be pregnant before April 2019 will also be excluded from the study as they have particularly different diets.

e. Are any of the participants likely to be vulnerable to coercion or undue influence?
Consider students, employees, terminally ill persons, or others who may have limited autonomy.

○ Yes
◉ No

If **Yes** --> Describe additional safeguards to protect participants' rights and welfare. ***Consider strategies to ensure voluntary participation.***

Section 15 - Participant Identification, Recruitment, & Selection

a. Provide evidence that you will be able to recruit the necessary number of participants to complete the study.

Belmont has approximate 20,000 loyalty card members, and similar studies have found that it is feasible to recruit up to 400 participants in a 1 week period.

b. Describe how potential participants will be identified (e.g., advertising, individuals known to investigator, record review, etc.). Explain how investigator(s) will gain access to this population, as applicable.

Participants will be recruited in person and using Belmont's social media (i.e. Facebook and email blast).

c. Describe the process that will be used to determine participant eligibility. ***Upload recruitment scripts to IRBNet.***

Participants will be firstly asked whether they are 18 years or older, whether they speak English, whether they purchase at least half of their weekly groceries at Belmont market, whether they are pregnant or plan to be pregnant before April 2019, and whether they are Belmont employee, which will be used to determine participant eligibility.

d. Describe the recruitment process; including the setting in which recruitment will take place.

Are copies of proposed recruitment materials (e.g., ads, flyers, website postings, recruitment letters, and oral/written scripts) uploaded onto IRBNet? (●) **Yes** ( ) **No**

Participants will be recruited in person and using Belmont's social media and asked to complete pre-screening questionnaire which clearly indicates the qualification requirements to participant in the study prior to randomization.

e. Explain how the process respects potential participants' privacy.

None of the data files will record any information that could identify a participant directly. Instead, each participant will be assigned a participant number, and only this number will be stored in the data as a way of coordinating the data. The participant numbers will not appear on the consent forms. All data files will initially be stored on the secure hard drives of the principal investigators, and/or co-investigators. Data extracted from the materials will kept on password-protected computers and identified solely by study number (and no participant names will be kept on that computer).

Section 16 - Incentives to Participate

Will participants receive compensation or other incentives (e.g., free services, cash payments, gift certificates, parking, classroom credit, travel reimbursement) to participate in the research study? ***Compensation plans should be pro-rated (not contingent upon study completion) and should consider participant withdrawals, as applicable.***

(●) Yes
( ) No

If **Yes** --> Describe the incentive, including the amount and timing of all payments.

All participants will receive a 5% discount on all grocery purchases after during the research study period (September 2018-April 2019), and also occasional weekly coupons. They will also receive $25 and $50 gift card if they complete the two follow-up 45-60 minutes surveys.

Section 17 - Alternatives to Study Participation (If Applicable)

Other than choosing not to participate, list any specific alternatives, including available procedures or treatments, that may be advantageous to the subject.

N/A

Section 18 - Informed Consent Process

Indicate the consent process(es) and document(s) to be used in the study. Check all that apply. ***Upload copies of documents and/or complete relevant appendices, as needed, to IRBNet.***

☐	Assent - Form	☐	Parental Permission - Form
☐	Assent - Verbal Script	☐	Parental Permission - Verbal Script --> Complete **Appendix M2**
☒	Informed Consent - Form	☐	Translated Consent/Assent- Form(s) --> Complete **Appendix J**
☐	Informed Consent - Verbal Script --> Complete **Appendix M2**	☐	Waiver or Alteration of Consent Process --> Complete **Appendix M1**
☐	Informed Consent - Addendum	☐	Waiver of Signed Consent --> Complete **Appendix M2**

a. Describe the consent process. Explain when and where consent will be obtained and how subjects and/or their legally authorized representatives will be provided sufficient opportunity (e.g., waiting period, if any) to consider participation. **Will a teach back process be employed?** ◯ Yes ◉ No

☐ N/A

Participants will complete a written informed consent in person with a research assistant before they complete the baseline survey at a time of their convenience between July and September 2018.

b. Explain how the possibility of coercion or undue influence will be minimized in the consent process.

☒ N/A

Project staff will be trained to go through the consent very carefully with the participant, fully explaining that participation is voluntary and that even if they agree to participate they can drop out of the study at any time.

c. Will any other tools (e.g., **quizzes, visual aids, information sheets**) be used during the consent process to assist participant comprehension?

◯ Yes --> **Upload copies of these tools to IRBNet**
◉ No

d. Will any other **consent forms** be used (e.g., for clinical procedures such as MRI, surgery, etc. and/or consent forms from other institutions)?

If Yes --> Indicate if another IRB has approved them and which one(s). Indicate that approval is pending if that is the case.

◯ Yes --> **Upload copies of these forms to IRBNet**
◉ No

Section 19 - Privacy of Participants

a. Describe the provisions to protect the privacy interests of the participants. ***Consider the circumstances and nature of information to be obtained, taking into account factors (e.g., age, gender, ethnicity, education level, etc.) that may influence participants' expectations of privacy.***

Participants will be assigned a study number unrelated to any identifying information, which will be used to identify participants throughout the study. Demographics, study number and name information will be kept in a folder that is separated from the study materials. All data analyses will be conducted using only participant study number.

b. Does the research require access to personally identifiable private information?

◯ Yes
◉ No

If Yes --> Describe the personally identifiable private information involved in the research. List the information source(s) (e.g., educational records, surveys, medical records, etc.).

Section 20 - Confidentiality of Data

a. Explain how information is handled, including storage, security measures (as necessary), and who will have access to the information. Include both electronic and hard copy records. **Methods for handling and storing data (including the use of personal computers and portable storage devices) must comply with university policies. Please include URI Building & Room Location. Note: paper copies of informed consent must be stored ON-CAMPUS.**

All data files will initially be stored on the secure hard drives of the principal investigators, and/or co-investigators. Data extracted from the materials will kept on password-protected computers and identified solely by study number (and no participant names will be kept on that computer). Data will be reported in summary format, and no names will be used. Only study personnel will have access to data. Personal identifiers will be removed from the final data set.

b. Explain if any personal or sensitive information that could be potentially damaging to participants (e.g., relating to illegal behaviors, alcohol or drug use, sexual attitudes, mental health, etc.) will be collected. Indicate when will data be destroyed and how.

☒ N/A

c. Will you be obtaining an **NIH Certificate of Confidentiality**?

○ Yes --> Upload a copy before you begin the research
◉ No

d. Explain any circumstances (ethical or legal) where it would be necessary to break confidentiality.

☒ N/A

e. Indicate what will happen to identifiable data at the end of the study. **Research data should be retained for a minimum of three years after final project closeout. (FIVE YEARS IF FDA STUDY)**

☐ Identifiable data will not be collected

☐ Identifiers will be permanently removed from the data and destroyed (resulting in de-identified data)

☒ Identifiable or coded/linked data will be retained and stored securely (as appropriate)

☐ Identifiable data will be retained and may be made public with participant consent (e.g., ethnographic research)

Section 21 - HIPAA Research Authorization

Will individually identifiable Protected Health Information (PHI) subject to the HIPAA Privacy Rule requirements be accessed, used, or disclosed in the research study?

- (●) No
- ( ) Yes --> Check all that apply:
  - ☐ Written Authorization --> Provide a copy of the Authorization Form
  - ☐ Partial Waiver (recruitment purposes only) --> ***Complete Appendix N***
  - ☐ Full Waiver (entire research study) --> ***Complete Appendix N***
  - ☐ Alteration (written documentation) --> ***Complete Appendix N***

Section 22 - Reasonably Anticipated Benefits

a. List the potential benefits that participants may expect as a result of this research study. State if there are no direct benefits to individual participants. ***Compensation is not to be considered a benefit.***

Participants will receive nutrition education and monetary incentives to improve overall diet quality.

b. List the potential benefits that society and/or others may expect as a result of this research study.

The results of the study will inform the development of the wellness initiatives which have the potential to promote population health on a broader scale.

Section 23 - Risks, Harms & Discomforts

a. Describe all reasonably expected risks, harms, and/or discomforts that may apply to the research. Discuss severity and likelihood of occurrence. As applicable, include potential risks to an embryo or fetus if a woman is or may become pregnant. ***Consider the range of risks, including physical, psychological, social, legal, and economic.***

There are no more than minimal risks, harms, or discomforts that apply to this research. The research does not require participants to behave any differently or expose them to anything that materially departs from their normal shopping experiences.

b. Describe how risks, harms, and/or discomforts will be minimized. ***If testing will be performed to identify individuals who may be at increased risk (e.g., pregnant women, individuals with HIV/AIDS, depressive disorders, etc.), address timing and method of testing; include how positive test results will be handled.***

There are no more than minimal risks or harms from participating in this research.

Section 24 - Monitoring

Does the research involve greater than minimal risk (i.e., are the harms or discomforts described in Section 22 beyond what is ordinarily encountered in daily life or during the performance of routine physical or psychological tests)?

○ Yes
◉ No

If **Yes** --> Describe the plan to oversee and monitor data collected to ensure participant safety and data integrity. Include the following:

- The information that will be evaluated (e.g., incidence and severity of actual harm compared to that expected);
- Who will perform the monitoring (e.g., investigator, sponsor, or independent monitoring committee);
- Timing of monitoring (e.g., at specific points in time, after a specific number of participants have been enrolled); and
- Decisions to be made as a result of the monitoring process (e.g., provisions to stop the study early for unanticipated problems).

Section 25 - Assessment of Risks & Benefits

Discuss how risks to participants are reasonable when compared to the anticipated benefits to participants (if any) and the importance of the knowledge that may reasonably be expected to result.
***Must be answered by all researchers**

Risks are minimal in this study, and do not go beyond the risk that consumers normally face when purchasing groceries in Belmont market. The potential improvement in dietary quality greatly outweighs the minimal risks.

Section 26 - Participant Costs/Reimbursements

a. List any potential costs participants (or their insurers) will incur as a result of study participation (e.g., parking, study drugs, diagnostic tests, etc.).	None
b. List any costs to participants that will be covered by the research study.	None

Assurance - Certifications and Endorsements by Principal Investigator

I agree to follow all applicable federal regulations, guidance, state and local laws, and university policies related to the protection of human subjects in research, as well as professional practice standards and generally accepted good research practices for investigators, including, but not limited to, the responsibilities described in the URI IRB policy.

☒ I verify that the information provided in this IRB Application for Human Subjects Research is accurate and complete.

☒ I verify that this IRB Application has been shared (on IRBNet) with the PI, all Co-investigators, and the Department Chair.

Print the form for your records

Print Form

22
