# Supplementary material for: Effect of Personalized Incentives on Dietary Quality of Groceries Purchased: A Randomized Crossover Trial
Source: JAMA Netw Open. 2021 Feb 10;4(2):e2030921. doi: 10.1001/jamanetworkopen.2020.30921 (PMC7876589; doi:10.1001/jamanetworkopen.2020.30921)
Supplement: Supplement 2. — eFigure. Change in Healthy Eating Index 2010 Scores Among 170 Smart Cart Study Participants With Valid Dietary Data eTable 1. Coupon Format Frequency in the Smart Cart Study eTable 2. Email Open Rate and Mean Shopping Trips per Week in the Smart Cart Study [file jamanetwopen-e2030921-s002.pdf]

Supplemental Online Content

Vadiveloo M, Guan X, Parker HW, et al. Effect of personalized incentives on dietary quality of groceries purchased: a randomized crossover trial. *JAMA Netw Open*. 2021;4(1):e2030921. doi:10.1001/jamanetworkopen.2020.30921

eFigure. Change in Healthy Eating Index 2010 Scores Among 170 Smart Cart Study Participants With Valid Dietary Data

eTable 1. Coupon Format Frequency in the Smart Cart Study

eTable 2. Email Open Rate and Mean Shopping Trips per Week in the Smart Cart Study

This supplemental material has been provided by the authors to give readers additional information about their work.

© 2021 Vadiveloo M et al. *JAMA Network Open.*

eFigure. Change in Healthy Eating Index 2010 Scores[a] Among 170 Smart Cart Study Participants With Valid Dietary Data[b]

	**Complete Case Analysis[c]** ($P_{treatment}$ =0.98, P_{period} =0.04; $P_{carryover}$=0.10)		**Multiple Imputation Analysis[d]** ($P_{treatment}$ = 0.14, P_{period} =0.03; $P_{carryover}$=0.09)	
	Initial intervention	Crossover intervention	Initial intervention	Crossover intervention
Group1<br>N<br>Mean (SD)<br>(95%CI)	 74<br>-1.83 (7.35)<br>(-3.53 to -0.13)	 74<br>-0.49 (6.59)<br>(-2.01 to 1.04)	 87<br>-1.68 (7.25)<br>(-3.35 to -0.25)	 87<br>-0.37 (6.62)<br>(-1.85 to 0.98)
Group2<br>N<br>Mean (SD)<br>(95%CI)	 65<br>-0.04 (8.44)<br>(-2.14 to 2.05)	 65<br>1.33 (7.22)<br>(-0.46 to 3.12)	 83<br>0.18 (8.41)<br>(-1.72 to 1.94)	 83<br>1.75 (7.33)<br>(0.07 to 3.30)

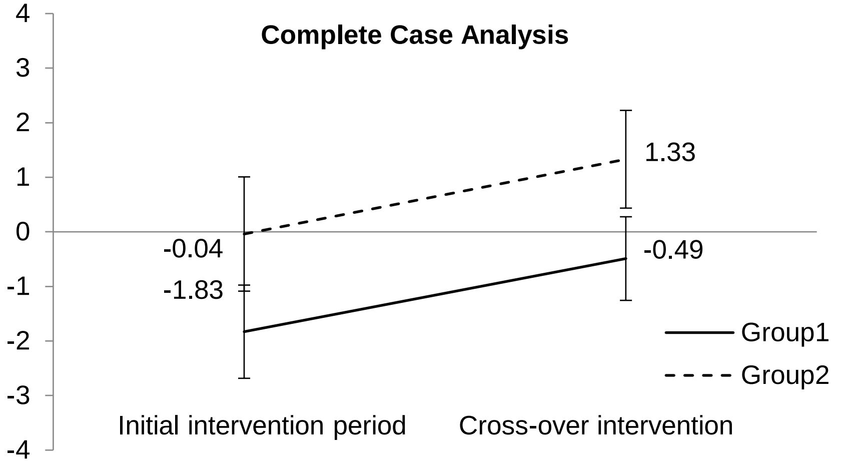

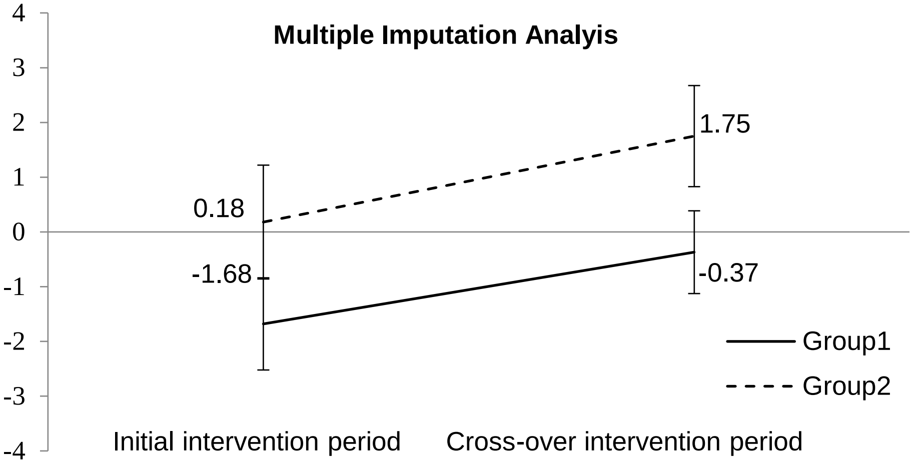

Data are presented as mean (SE) with SEs denoted with error bars
[a]Change scores for the initial intervention period: Midpoint HEI-10 - Baseline HEI-10; Change scores for the cross-over period: Endpoint HEI-10 - Midpoint HEI-10
[b]Group 1: The group that was randomized to receive the treatment in the initial intervention period followed by an active control in the second intervention period. Group 2: The group that was randomized to receive the active control in the initial intervention period followed by the treatment in the second intervention period.
[c]170 participants were left after excluding implausible values. Of the 170 participants, 31 had missing scores either at midpoint or at study completion, leaving a total number of 139 unique participants included in the complete case analysis.
[d]Multiuppple imputation models used age, body mass index, and smoking status as auxilliary variables associated with missingness

© 2021 Vadiveloo M et al. *JAMA Network Open.*

eTable 1. Coupon Format Frequency in the Smart Cart Study

	**Total**<br>**N**	**Buy-one-get-one**<br>**N**<br>**%**	**Dollar_off ($2 - $10 off)**<br>**N**<br>**%**	**Percent_off (20% - 50% off)**<br>**N**<br>**%**	**Free item**<br>**N**<br>**%**
Initial intervention period	2880	274<br>9.5%	431<br>15.0%	2175<br>75.5%	0<br>0%
Crossover intervention period	2576	112<br>4.3%	499<br>19.4%	1950<br>75.7%	15<br>0.6%
**Number of Unique Coupons**					
Initial intervention period	122	7<br>5.7%	23<br>18.9%	92<br>75.4%	0<br>0%
Crossover intervention period	141	8<br>5.7%	21<br>14.9%	111<br>78.7%	1<br>0.7%

© 2021 Vadiveloo M et al. *JAMA Network Open.*

eTable 2. Email Open Rate and Mean Shopping Trips per Week in the Smart Cart Study

Email Open times (per week)				
	**Initial intervention period**	**Cross-over intervention period**	**Overall**	
Group 1 (AB)	2.67 (3.61)	1.88 (2.05)	2.23 (3.06)	**2.28(2.53)**
Group 2 (BA)	2.08 (1.56)	2.30 (2.07)	2.24 (1.87)	
Overall	2.43 (2.87)	2.12 (2.12)		
**Shopping trips (per week)**				
Group 1 (AB)	1.64 (0.92)	1.62 (1.00)	1.63 (0.96)	**1.61(1.01)**
Group 2 (BA)	1.59 (1.03)	1.58 (1.09)	1.59 (1.06)	
Overall	1.61 (0.97)	1.61(1.04)		

© 2021 Vadiveloo M et al. *JAMA Network Open.*
