# Supplementary material for: Effect of Personalized Incentives on Dietary Quality of Groceries Purchased: A Randomized Crossover Trial
Source: JAMA Netw Open. 2021 Feb 10;4(2):e2030921. doi: 10.1001/jamanetworkopen.2020.30921 (PMC7876589; doi:10.1001/jamanetworkopen.2020.30921)
Supplement: Supplement 3. — Data Sharing Statement [file jamanetwopen-e2030921-s003.pdf]

Data Sharing Statement

Vadiveloo. Effect of Personalized Incentives on Dietary Quality of Groceries Purchased. *JAMA Netw Open*. Published February 10, 2021. doi:10.1001/jamanetworkopen.2020.30921

Data
Data available: No
